# Effects of Physical Exercise on Anxiety and Depression of People with Fibromyalgia: Umbrella Review of Systematic Reviews and Meta-Analyses

**DOI:** 10.3390/jfmk11020193

**Published:** 2026-05-12

**Authors:** Nuria Pérez-Romero, Annais Rubilar-Barrera, Constanza Carolina Salinas-Parada, Karen Navarrete-Valenzuela, Valentina Paz Vera-Espinoza, Oscar Núñez, Enrique Cerda-Vega

**Affiliations:** 1 Exercise and Rehabilitation Sciences Institute, Postgraduate, Faculty of Rehabilitation Sciences, Universidad Andres Bello, Santiago 7591538, Chile; nuria.perez@unab.cl; 2 Exercise and Rehabilitation Sciences Institute, School of Physical Therapy, Faculty of Rehabilitation Sciences, Universidad Andres Bello, Santiago 7591538, Chile; 3 Exercise and Rehabilitation Sciences Institute, Faculty of Rehabilitation Sciences, PhD in Rehabilitation Sciences, Universidad Andres Bello, Santiago 7591538, Chile; o.nuezdiaz@uandresbello.edu

**Keywords:** mental health, chronic pain, emotions, physical activity, well-being

## Abstract

**Background:** Fibromyalgia is a chronic nociplastic pain condition often accompanied by mental health comorbidities, with anxiety and depression being the most prevalent. The objective of this umbrella review is to analyze the effects of physical exercise on anxiety and depression symptoms in individuals with fibromyalgia. **Methods**: Following Cochrane and PRIOR guidelines, a systematic search was conducted in PubMed, Web of Science, Scopus, and CINAHL Complete up to 28 August 2025. Systematic reviews with or without meta-analyses that evaluated physical exercise interventions in adults with fibromyalgia and reported anxiety or depressive symptom outcomes were included. Risk of bias was assessed with AMSTAR-2; overlap was evaluated using MOoR and CCA. **Results**: Fourteen reviews (eight meta-analyses, three systematic reviews, two meta-analyses treated as descriptive, and one network meta-analysis) were included, synthesizing 98 randomized controlled trials (RCTs) with 4325 participants (in the 12 reviews that provided data). The majority of the patients were women and people aged between 10 and 65. Regarding anxiety, five of seven reviews reported significant improvements. Aquatic exercise showed the greatest effect (SMD = −1.14). Regarding depression, eight of 11 reviews reported significant benefits. Aquatic exercise again stood out with the highest effect (SMD = −1.18). Adherence varied between 64% and 97%. Methodological quality according to AMSTAR-2 showed considerable heterogeneity. **Conclusions**: Physical exercise, especially aerobic and aquatic modalities, may support the reduction of symptoms of anxiety and depression in people with fibromyalgia. These findings support its inclusion in rehabilitation programs, although methodological and prescription variability suggests caution in interpreting optimal parameters. PROSPERO-ID: CRD42024590799.

## 1. Introduction

According to the International Association for the Study of Pain (IASP) in 2020, “pain is an unpleasant sensory and emotional experience associated with or resembling that associated with actual or potential tissue damage” [1] and can be classified as nociceptive, neuropathic, and nociplastic pain [2,3]. Nociplastic pain is a key player in chronic pain conditions, such as fibromyalgia (FM) [3]. FM is considered the third most common musculoskeletal condition involving widespread chronic pain that lasts beyond three months [4,5] affecting between 0.4 and 8.8% of various populations worldwide, with a global prevalence between 2 and 3% [6]. Specifically, prevalence is higher in females (4.2%) than in males (0.2%), with a 21:1 female/male ratio [7]. Despite several mechanisms appearing to be involved in the development of pain in FM, central sensitization (CS) [8] is the most plausible mechanism. However, pain is not the only symptom present in FM, and others include fatigue, sleep disturbances, stiffness, autonomic disturbances, regional pain syndromes, hypersensitivity to external stimuli, cognitive dysfunctions and psychiatric symptoms such as anxiety and depression [6].

Mental health is understood as a dynamic and heterogeneous process that manifests differently in each person; in this sense, the World Health Organization defines it as a “state of physical, mental, emotional, and social well-being, determined by the individual’s interaction with society” [9]. Within this framework, it is important to distinguish between mental disorders, as diagnostic categories established by the Diagnostic and Statistical Manual of Mental Disorders [10], and symptomatic manifestations, which may occur in isolation or sub clinically, without meeting diagnostic criteria, and are often assessed using questionnaires. Depressive mood and anxiety, addressed as symptoms, are characterized respectively by a persistent low mood, reduced energy, and decreased interest or pleasure in usual activities (depressive symptoms), as well as a state of physiological arousal associated with hypervigilance, restlessness, or tension (anxious symptoms). At the neurobiological level, these manifestations have been linked to alterations in various neurotransmission systems, including dopamine in the mesocortical and mesolimbic pathways, serotonin, and norepinephrine, as well as with the activation of the sympathetic nervous system mediated by the release of catecholamines and increased corticotropin-releasing hormone tone, processes involved in the regulation of alertness and stress response [11,12].

In individuals living with chronic pain, mental health comorbidities are common [13] and exhibit a high prevalence in individuals with fibromyalgia [14,15]. Specifically, depression and anxiety are most common in individuals with fibromyalgia [16], with a prevalence up to 88.3% for depression [17] and 77.5% for anxiety [18]. Pharmacological (antidepressants, antiepileptics and others) and non-pharmacologic interventions are available, such as education, cognitive/psychological therapy, diet/supplements, adjunct modalities (e.g., acupuncture and transcutaneous electrical nerve stimulation), and exercise [19]. Exercise is defined as “physical activity that is planned, structured, repetitive, and purposive in the sense that improvement or maintenance of one or more components of physical fitness is an objective” [20]. Exercise has been recommending to a “strong degree” by the European League Against Rheumatism (EULAR) for the management of symptoms in FM [21]. This strategy is very important because exercise has been proposed as a non-pharmacological treatment [21] not only to decrease pain [22,23,24], but also for mental health improvement, such as depression [22,23,24] and anxiety [25,26]; specifically, resistance training [27] and aerobic exercise [28] have been recommended. The mechanisms by which exercise decreases depression and anxiety involve hormone regulation, immune function, inflammation, brain structures, and neurotransmitters [29,30].

Furthermore, the benefits of physical exercise in fibromyalgia extend beyond symptom management, as it is also positively associated with self-efficacy, central neuromodulation and a reduction in the inflammatory response [31,32]. Previous reviews have reported significant improvements, with moderate effect sizes, in pain intensity [26,33], fatigue [34], sleep [33,34], and the physical and mental dimensions of quality of life [26,35]. These benefits are consistent across various modalities: strength training stands out for its ability to reduce fatigue and improve mood, whilst mind–body therapies (such as yoga, tai chi and qigong) achieve a systemic reduction in anxiety and depression through the activation of the endogenous opioid system [31,36]. However, findings across reviews show high heterogeneity due to variations in intervention styles, follow-up timeframes and the lack of standardized protocols [36,37]. Unlike previous syntheses that focus on a single modality or symptom, this study critically integrates the highest-level evidence to compare the relative efficacy of different forms of exercise.

Only two umbrella reviews have examined the effects of exercise on fibromyalgia [38,39]; however, neither of them focus on mental health. Furthermore, the growing number of systematic reviews and meta-analyses on exercise interventions for fibromyalgia has yielded inconsistent results across different exercise modalities, outcomes and methodological approaches, making it difficult for clinicians and researchers to obtain a comprehensive overview of the evidence. Important questions also remain regarding which exercise modalities may be most beneficial in terms of reducing symptoms of anxiety and depression, and which intervention characteristics may be associated with better outcomes. Therefore, conducting an umbrella review could be useful for synthesizing and comparing the results of existing reviews, assessing the consistency of findings, identifying common characteristics of interventions, and providing a high-level summary of the evidence regarding the effects of exercise on symptoms of anxiety and depression in people with fibromyalgia.

Consequently, an important question remains unresolved: what are the effects of physical exercise on the mental health of individuals with fibromyalgia, specifically in relation to symptoms of anxiety and depression? Therefore, the main objective of this umbrella review was to analyze the effects of exercise on symptoms of depression and anxiety in individuals with fibromyalgia. A secondary objective was to identify the exercise modalities and intervention characteristics associated with the outcomes, including frequency, intensity, time (duration), and type of exercise (FITT principles). This research could be beneficial to rehabilitation professionals regarding program design and strategies for improving the mental health of individuals with fibromyalgia. This review may provide clinically relevant guidance for rehabilitation professionals by supporting evidence-informed decisions regarding the selection, design, and implementation of exercise-based strategies aimed at improving psychological well-being in individuals with fibromyalgia. In particular, the findings may help clinicians consider which types of exercise have shown the most promising effects and how exercise may be incorporated into multidisciplinary symptom management programs.

## 2. Materials and Methods

In this study, an overview of reviews (also known as umbrella review) was conducted. To this end, the guidance provided by the Cochrane Handbook for Reviews of Reviews was followed [40]. In addition, the Preferred Reporting Items for Overviews of Reviews (PRIOR) guidelines were applied [41] to ensure the transparency, systematization, and methodological rigor of the review, with all items of the corresponding checklist being fulfilled. A previous protocol was registered in PROSPERO ID: CRD42024590799.

### 2.1. Data Sources and Search Strategy

Four electronic databases were used (PubMed, Web of Science, Scopus, and CINAHL Complete). The initial search was conducted on 5 August 2024, and an updated search was subsequently performed through 28 August 2025 to identify any additional eligible reviews published after the original search date. No language restrictions were applied. Only peer-reviewed publications meeting the predefined eligibility criteria were considered for inclusion. Medical Subject Headings (MeSH) terms and keywords were combined using Boolean operators. The full search strategy is available in Table 1.

### 2.2. Eligibility Criteria

The studies included in this review met the below eligibility criteria (Table 2). No temporal or language restrictions were applied. It should be noted that structured exercise was defined as a planned and intentional exercise program that included at least some defined characteristics (e.g., type of exercise, frequency, duration, intensity, progression or supervision), even when not all components were reported in full. Conversely, studies relating to physical activity, such as walking, were excluded when the intervention consisted solely of a general recommendation or advice to stay active (e.g., ‘go for a walk’) without a clearly described exercise program.

### 2.3. Study Selection

All search results were uploaded to Rayyan web version (https://www.rayyan.ai/; accessed on 28 August 2025) for duplicate removal and screening of titles, abstracts, and full texts. Four authors (A.R.-B., C.C.S.-P., K.N.-V., and V.P.V.-E.) independently and equally screened titles and abstracts against the eligibility criteria. The remaining articles were then read in full independently by two author groups (A.R.-B. and K.N.-V.) and (C.C.S.-P. and V.P.V.-E.). The information obtained by both groups was compared to identify potential disagreements, which were resolved through consultation with a fifth author (N.P.-R.). The entire process was reviewed by an additional author (E.C.-V.). The article selection process was reported using a flow diagram from the Preferred Reporting Items for Systematic Reviews and Meta-Analyses (PRISMA) guidelines [44].

### 2.4. Data Extraction

Data extraction was performed independently by the two author groups (A.R.-B. and K.N.-V.; C.C.S.-P. and V.P.V.-E.). The included studies were divided equally between the groups, such that each group extracted data from half of the selected studies. Extracted data were recorded in an Excel 365 spreadsheet and subsequently incorporated into the final manuscript. To ensure consistency and accuracy, the extracted information from both groups was cross-checked, and any discrepancies were resolved through discussion and consultation with a fifth author (N.P.-R.). The entire data extraction process was reviewed by an additional author (E.C.-V.).

### 2.5. Data Item

Data was extracted according to the characteristics of each review, including the number of primary studies included and the review’s search strategies (name and number of databases searched, and the dates of the search when available). In addition, population characteristics were extracted (sample size, age, and gender). Information on the intervention and comparator was also collected (type of exercise and comparators analyzed, exercise intensity, frequency, number of sessions, total intervention duration, and adverse events). Outcomes included mental health variables such as anxiety and depression, including the total number of experimental and control groups in each review and their respective results when reported quantitatively. For reviews that included meta-analyses, detailed quantitative data were extracted, including the meta-analytic model applied (fixed-effect or random-effects), the effect size metric used (e.g., standardized mean difference, Cohen’s d, or Hedges’ g), pooled effect estimates for anxiety and/or depression outcomes, and their 95% confidence intervals. Extracted effect sizes were subsequently classified as small, moderate, or large.

### 2.6. Risk of Bias Assessment

Risk of bias was independently and comprehensively assessed by the two author groups using A Measurement Tool to Assess systematic Reviews (AMSTAR-2; [45]). Disagreements were resolved through discussion and reviewed by the fourth and fifth authors. AMSTAR-2 is a critical appraisal instrument for systematic reviews that include both randomized and non-randomized studies and consists of 16 critical and non-critical items with response options of yes (✓), no (x), or partial yes (-). The results were reported to enhance transparency but were not used as criteria for study exclusion. Risk of bias was assessed only at the level of the included systematic reviews, without re-assessing the primary studies.

### 2.7. Overlap of Primary Studies

As this was an umbrella review, the final included reviews could contain overlapping primary studies, which may have influenced the conclusions of the present review. To avoid duplication of information as much as possible, the MOoR Framework [46] was followed. In this case, an analysis was performed of all studies that met the eligibility criteria. (1) When a review had all primary studies repeated with other reviews, only one of them was included, prioritizing meta-analyses over systematic reviews, studies that separate their analysis by type of exercise over those that do not, and the study with the highest number of primary studies in that order; (2) although all studies were reviewed in full, relevant data were added if not mentioned in the previous ones, but without duplicating quantitative values; (3) primary studies were not excluded based on the assessment of risk of bias; and (4) the Corrected Covered Area (CCA) of the articles finally included in the synthesis was shown, in addition to detailing the entire process and selection decisions in the results.

Therefore, the degree of overlap was described using a citation matrix to visualize the extent of overlap of primary studies across the included systematic reviews. In this matrix, rows represented all primary studies and columns represented the names of all final reviews included in the study. Each primary study was marked with an “x” at its first occurrence. From this matrix, the total number of publications N and R were obtained (N, calculated as the sum of all cells marked as “1,” including duplicates; R, defined as the number of unique, non-duplicated primary studies), as well as the total number of columns (C), corresponding to the total number of included reviews. Using these values, the CCA was calculated. Overlap was then classified as slight (CCA 0–5%), moderate (CCA 6–10%), high (CCA 11–15%), or very high (CCA > 15%) [47].

$$CCA=\frac{N-r}{r\times c-r}\times 100$$

### 2.8. Data Synthesis of the Included Reviews

The extracted data were synthesized descriptively and organized into thematic categories based on mental health outcomes, specifically anxiety and depression. Results were summarized in tables and grouped according to these outcome domains. A comparative analysis was conducted by examining the direction and magnitude of the reported effect sizes across reviews, allowing for the identification of similarities and differences between studies and the exploration of potential sources of heterogeneity. As most reviews did not explicitly define thresholds for small, moderate, or large effects, effect size magnitudes were interpreted according to Cohen’s guidelines [48], whereby standardized mean differences of approximately 0.20, 0.50, and 0.80 were considered small, moderate, and large effects, respectively.

The authors declare that, during the preparation of this review manuscript, generative artificial intelligence tools (ChatGPT-5 and NotebookLM) were used to support the refinement of academic language, improve clarity of expression, and assist in the organization and grouping of information extracted from the literature. All outputs generated by these tools were used solely to cross-check and verify information previously compiled by the authors and were subsequently reviewed and edited by the authors. These tools were not used to generate original scientific content, data, analyses, or interpretations, nor to influence the study design or conclusions. Full intellectual responsibility for the content of the manuscript rests with the authors.

## 3. Results

### 3.1. Selection

**Figure 1:**
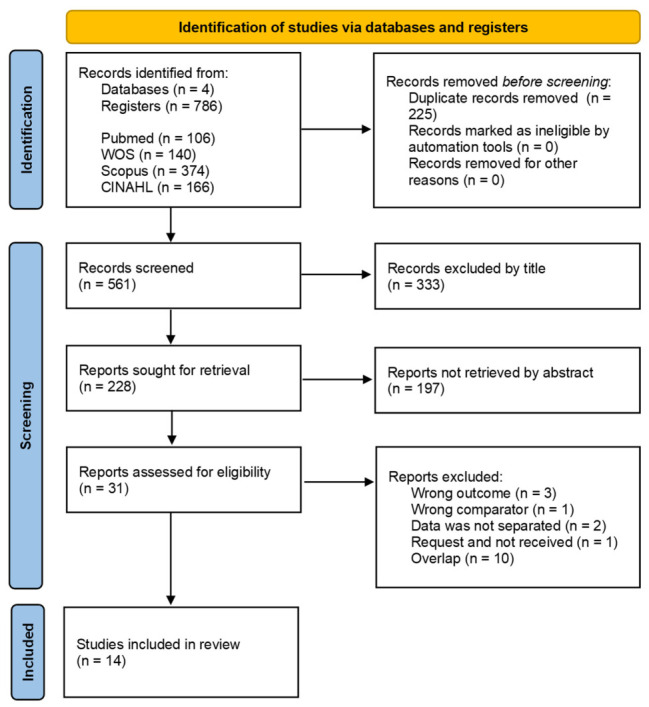
Selection flow diagram by PRISMA.

Figure 1 shows the study selection process. A total of 786 records was identified through databases; after removing 225 duplicates, 561 unique records proceeded to the screening phase. During the screening of titles and abstracts, 333 and 197 records were excluded for not meeting the criteria, leaving 31 articles for full-text evaluation. Finally, after excluding 17, 14 studies were included in the review. Supplementary Materials S1 provides a detailed list of the full-text studies that were excluded and the reasons for their exclusion.

### 3.2. Study Characteristics

Table 3 summarizes the characteristics of the 14 reviews included (eight meta-analyses, three systematic reviews, two meta-analyses treated as systematic reviews, and one network meta-analysis). Two meta-analyses were analyzed as systematic reviews because they did not stratify results by fibromyalgia in the quantitative analysis. The number of databases used to perform the searches ranged from 3 to 9 databases per review, including studies from January 1995 to September 2024. The number of randomized controlled trials included ranged from one to 25 per review, with a total of 98 RCTs across all included reviews. When reported, total sample sizes ranged from 20 to 668 participants; across the 12 reviews that provided sample size data, this corresponded to an overall total of 4325 participants. Among these, nine reviews reported allocation by group, comprising 2106 participants in experimental groups and 2036 participants in control groups, while five reviews did not report sample size information. Gender information is presented in 10 reviews, of which seven include only women, two include men and women, and one includes men, women, and unreported participants, while four do not report this data. Finally, age is reported in 11 reviews, with ranges from 10 to 65 years or a mean of 51 years and remains unreported in three reviews.

### 3.3. Results from Anxiety

Across the seven reviews that reported anxiety outcomes, exercise interventions were delivered 1 to 9 times per week over 4 to more than 48 weeks, with intensities ranging from low to high or expressed as 65–75% of HRmax when specified (Table 4). A wide variety of exercise modalities were evaluated, including aerobic exercise (land- and water-based), aquatic exercise, dance, walking/jogging, yoga/mind–body exercise, stretching, resistance training, circuit-based exercise, movement techniques, and Qigong, compared mainly with usual care, wait-list controls, no intervention, education, or passive/active comparators. In total, five of the seven reviews reported significant improvements in anxiety, either through meta-analytic estimates or descriptive synthesis, while two reviews reported mixed or non-significant results depending on intervention type or intensity progression. Meta-analyses showed effect sizes ranging from WMD −0.77 to SMD −1.14, with aquatic exercise consistently demonstrating the largest effects on anxiety, whereas land-based aerobic and multicomponent programs showed small-to-moderate benefits. Anxiety was assessed using multiple validated instruments, most frequently STAI, HADS-A, FIQ-A, BDI, AIMS-A and MHI, reflecting substantial heterogeneity in outcome measurement. Adherence rates, when reported, ranged from 72% to 97%, drop-out rates varied between 0% and 50%, and adverse effects were rarely reported, with only isolated cases of pain or injury noted.

### 3.4. Results from Depression

Across the 11 reviews reporting depression outcomes (Table 5), exercise interventions were delivered 1 to 9 times per week over intervention periods ranging from 3 to 32 weeks, with intensities described as low-to-high, 65–75% HRmax, or 40–80% RPM when specified. The interventions included aerobic exercise (land-based and aquatic), strength training, stretching, multicomponent therapeutic exercise, dance (e.g., Zumba), and mind–body exercises such as Yoga, Tai Chi, Qigong, Baduanjin, Wuqinxi and Yijinjing, compared mainly with usual care, no intervention, education, stretching, relaxation, aerobic exercise, or wait-list controls. In terms of outcomes, eight of the 11 reviews reported significant improvements in depression for at least one exercise modality, two reported mixed or non-significant results, and one found no differences between groups. Meta-analytic estimates, when conducted, showed effect sizes ranging from SMD −0.43 to −1.18, with aquatic exercise and hydrotherapy showing the largest effects, followed by strength training and mind–body interventions, while some Tai Chi and Qigong modalities did not reach statistical significance. Depression was assessed using a wide range of validated instruments, most frequently the BDI/BDI-II, HADS-D, CES-D, FIQ-D and HAMD. Adherence rates, when reported, ranged from 64% to 97%, drop-out rates varied from 0% to 50% or were explicitly reported as 101 participants in one review, and adverse effects were infrequently reported, consisting mainly of mild musculoskeletal pain or inflammation, with several reviews reporting no adverse events.

### 3.5. Risk of Bias

**Figure 2:**
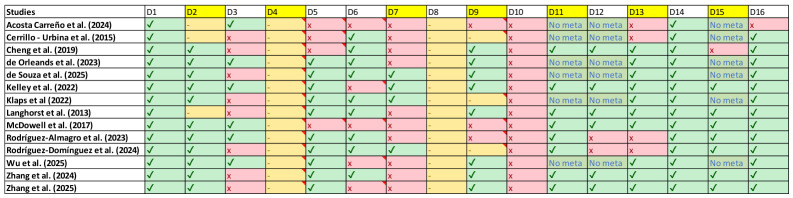
AMSTAR-2 results by articles [25,26,31,32,33,34,35,36,37,49,50,51,52,53].

Figure 2 shows the methodological quality assessment of the included reviews using domains D2 to D15 of the AMSTAR-2 instrument, where each row corresponds to a review and each column to a specific domain; the symbols indicate whether the criterion is met (✓), not met (x), unclear (-) or not applicable/no meta-analysis was performed (‘No meta’). Overall, it can be seen that most reviews adequately meet the domains related to the clear formulation of the question, the literature search and the description of the included studies, while there are recurring weaknesses in key domains such as the justification of exclusions, the assessment of risk of bias, the consideration of risk of bias in the interpretation of results and, where appropriate, the justification and adequate performance of meta-analysis. Reviews without meta-analysis correctly show statistical domains as not applicable, but still have relevant methodological limitations, indicating considerable heterogeneity in the overall quality of the reviews and justifying a cautious interpretation of their conclusions.

**Figure 3:**
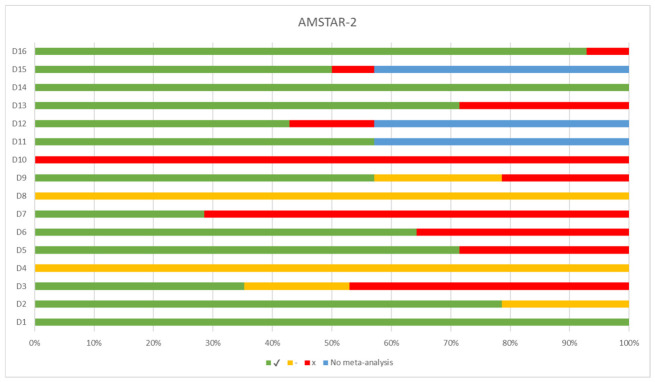
AMSTAR-2 results by domain.

Figure 3 shows the risk of bias by domain. Compliance was high in D1 (PICO) (100%; 14/14) and D16 (conflicts of interest) (93%; 13/14), as well as in D2 and D14 (79%; 11/14) and in D5 and D13 (71%; 10/14). In contrast, critical weaknesses were identified in D10 (sources of funding for primary studies), which was not complied with by any review (100%; 14/14), and in D7 (list of excluded studies), with 71% (10/14) non-compliance.

### 3.6. Overlap

Based on the final sample of 14 included reviews, after excluding studies during the inclusion phase according to the Moore framework, 76 unique primary studies (R) and 110 total study occurrences across reviews (N) were identified. The resulting CCA was 3.44%. According to the interpretative thresholds, this value represents a low overlap (0–5%) among reviews, indicating that the overall level of duplication of primary studies across the included reviews was low. The matrix can be found in Supplementary Materials S1.

$$CCA=\frac{N-r}{r\times c-r}\times 100=\frac{110-76}{76\times 14-76}\times 100=3.44\%$$

## 4. Discussion

The aim of this study was to analyze the effects of physical exercise on the mental health of individuals with fibromyalgia, specifically in relation to symptoms of anxiety and depression.

The results show that, in the case of anxiety, aerobic physical exercise, particularly aquatic exercise, was the most studied modality and the one that presented the highest effect sizes in favor of the intervention in reducing symptoms, with effects ranging from moderate to large. This benefit can be explained, at least in part, by the effect of aerobic exercise on the serotonergic system, since serotonin or 5-hydroxytryptamine (5-HT) is a key neurotransmitter in the regulation of mood, sleep, appetite, and motor control [54], and plays a central role in modulating anxiety [55]. In this regard, Cordeiro et al. (2017) [56] propose that physical exercise modulates the interaction between the dopaminergic and serotonergic systems through neuroplasticity mechanisms. Mind–body interventions, such as yoga, showed more heterogeneous results, with less consistent but potentially relevant benefits, possibly due to the combination of movement, controlled breathing, and mindfulness, which could contribute to stress reduction and nervous system regulation [57,58]. However, the effects of exercise on anxiety seem to depend on the type of intervention, intensity, duration, and characteristics of the population, which explains the variability observed between studies [59].

On the other hand, the results indicate that, in relation to depression, the effect of physical exercise is heterogeneous, although generally favorable, with consistent benefits observed in various modalities. The synthesized evidence shows that aquatic exercise has the greatest effect size, followed by strength training and some mind–body interventions, while modalities such as yoga and Tai Chi show variable results, with moderate or non-significant effects depending on the specific type of intervention and the comparator used. These effects can be explained by various physiological mechanisms, as physical exercise is associated with a reduction in neuroinflammation, a process involved in depression, through the decrease in pro-inflammatory markers such as TNFα and interleukins IL-1β, IL-6 and IL-2R [60]. Exercise also improves cardiorespiratory function, increasing brain oxygenation and promoting cognitive and emotional processes relevant to the reduction in depressive symptoms [61]. Finally, given that depression is modulated by social factors, interventions carried out in group settings or with social support can provide additional benefits, strengthening the sense of belonging and social connection, which could enhance the effects of exercise on mental health [62].

Regarding the frequency, intensity, time and time (FITT) of exercise, the results of this review indicate that the most effective interventions for reducing anxiety and depression mainly correspond to structured aerobic exercise, especially when performed at moderate intensities and on a regular basis. Therefore, beneficial effects were commonly reported in programs delivered 1–3 times per week over approximately 8–24 weeks, although considerable heterogeneity in dosage remains across studies. The available evidence does not allow for a definitive consensus on the optimal FITT, as while some studies did not observe significant reductions in depression with low- or high-intensity resistance exercise in people with fibromyalgia [63], other studies have reported benefits on anxiety and depression symptoms with moderate- and high-intensity interventions, particularly in aerobic or multicomponent programs [60,64].

In terms of adverse effects, the review shows that exercise, especially aerobic exercise, is generally safe, although some studies reported mild and transient side effects, such as muscle pain or fatigue, which did not require additional intervention. Considering that the mental health benefits far outweigh these minor effects, regular exercise in controlled and supervised settings is recommended, with a gradual progression in intensity to minimize discomfort and promote adherence [65,66].

Following Cohen’s conventional criteria, most of the modalities analyzed produced small-to-moderate reductions on anxiety (SMD: −0.28 to −0.77) and decreases in depression (SMD: −0.43 to −0.54), whereas aquatic exercise showed reductions of greater magnitude (SMD = −1.14 and −1.18, respectively), although these derive from a limited number of studies, which constrains the generalizability of these findings. It should be noted that although the effect sizes are small-to-moderate, their clinical relevance should not be underestimated. In the context of complex conditions and multifactorial interventions such as exercise, small changes in magnitude can translate into relevant functional benefits; therefore, their interpretation must consider the context of a chronic and difficult-to-manage condition like fibromyalgia. Some mind–body modalities did not reach statistical significance or yielded effects in the small range, suggesting caution when recommending these interventions as standalone strategies for the management of affective symptoms. Taken together, both the clinically meaningful magnitude of the expected benefit and the safety profile of each modality should guide an individualized, supervised prescription with gradual progression in intensity.

This review has significant limitations. First, the variability and incomplete reporting of the FITT in several studies prevent accurate comparisons between interventions and limit the identification of dose–response relationships. Second, in some studies, the absence of a formal clinical diagnosis of anxiety or depression makes it difficult to draw clear conclusions about the impact of exercise on these disorders, as symptoms are assessed rather than clinical pictures. Furthermore, methodological heterogeneity and the limited number of primary studies in certain modalities restrict the generalization of results. The heterogeneity observed across included reviews in terms of exercise modality, intervention dose, participant characteristics, outcome measures, and methodological quality should be considered when interpreting the findings, as it may influence the consistency, comparability, and generalizability of the reported effects. Therefore, it is recommended that future research include standardized psychological assessments, report in detail the frequency, intensity, type, and duration of exercise, and develop longitudinal analyses to explore long-term effects. Additionally, some included reviews did not consistently report key participant characteristics (e.g., age and sex/gender), which limits the ability to assess the applicability of findings across different subgroups and may reduce the generalizability of the results. Finally, it is essential to consider the proper diagnosis of fibromyalgia and its psychological comorbidities, which are often underdiagnosed, as these can influence both the effectiveness of exercise and the perception and adherence of participants, and condition the interpretation of the impact of exercise on mental health.

## 5. Conclusions

In summary, the results suggest that different types of physical exercise, mainly aerobic exercise, aquatic exercise, strength training, and some mind–body interventions, may be associated with reductions in anxiety and depression symptoms in people with fibromyalgia. Across the included reviews, beneficial effects were commonly observed in programs delivered 1–3 times per week, with many interventions lasting between 8 and 24 weeks, although considerable variability in frequency, duration, intensity, and modality was identified. These findings provide an updated synthesis of the available evidence that may help inform clinical decision-making regarding the incorporation of exercise as part of the approach to treating symptoms in this population. However, these results should be interpreted with caution due to the substantial heterogeneity observed in the characteristics of the interventions (frequency, intensity, type, and duration), in the assessment instruments used, and in the variable risk of bias of the reviews analyzed, which limits the possibility of establishing conclusions about the differential effect of each type of exercise or of precisely defining the optimal prescription parameters for its application in clinical practice. It remains difficult to determine the differential effect of each exercise modality or to establish precise optimal prescription parameters for routine clinical practice. Further high-quality research is needed to refine exercise recommendations according to participants characteristics and symptom profiles.

## Figures and Tables

**Table 1:** Search strategy on each database.

Database	Search Strategy
PubMed	(((((“mental health”[Title/Abstract]) OR (“mental care”[Title/Abstract])) OR (anxiety[Title/Abstract])) OR (depression[Title/Abstract])) AND ((fibromyalgia[Title/Abstract]) OR (fibrositis[Title/Abstract]))) AND ((((((“physical exercise”[Title/Abstract]) OR (exercise[Title/Abstract])) OR (“physical activity”[Title/Abstract])) OR (sport[Title/Abstract])) OR (yoga[Title/Abstract])) OR (pilates[Title/Abstract])) Filters: Meta-Analysis, Review, Systematic Review
Web of Science	16. #13 AND #14 AND #15; 15. #7 OR #8 OR #9 OR #10 OR #11 OR #12; 14. #3 OR #4 OR; #5 OR #6; 13. #1 OR #2; 12. TS=(pilates); 11. TS=(yoga); 10. TS=(sport); 9. TS=(“physical activity”); 8. TS=(“physical exercise”); 7. TS=(exercise); 6. TS=(depression); 5. TS=(anxiety); 4. TS=(“mental care”); 3. TS=(“mental health”); 2. TS=(fibrositis); 1. TS=(fibromyalgia) Filters: review articles
Scopus	((TITLE-ABS-KEY(fibromyalgia)) OR (TITLE-ABS-KEY(fibrositis))) AND ((TITLE-ABS-KEY(“mental health”)) OR (TITLE-ABS-KEY(“mental care”)) OR (TITLE-ABS-KEY(depression)) OR (TITLE-ABS-KEY(anxiety))) AND ((TITLE-ABS-KEY(exercise)) OR (TITLE-ABS-KEY(“physical activity”)) OR (TITLE-ABS-KEY(sport)) OR (TITLE-ABS-KEY(yoga)) OR (TITLE-ABS-KEY(pilates)) OR (TITLE-ABS-KEY(“physical exercise”))) AND (LIMIT-TO (DOCTYPE,“re”))
CINAHL Complete	(AB fibromyalgia OR AB fibrositis) AND (AB “mental health” OR AB “mental care” OR AB anxiety OR AB depression) AND (AB exercise OR AB “physical activity” OR AB “physical exercise” OR AB sport OR AB yoga OR AB pilates)

**Table 2:** Eligibility criteria.

PICOT	Inclusion	Exclusion
Population	Female and male participants diagnosed with fibromyalgia according to ICD-11 or any of its previous versions.	Participants diagnosed with severe mental disorders such as schizophrenia, as well as those with chronic non-communicable diseases such as dyslipidemia, hypertension, and diabetes.
Intervention	Both acute and chronic physical exercise, such as yoga, Pilates, aquatic therapy, resistance training, among others.	No type of exercise was excluded; however, studies examining physical activity (e.g., walking) without a clearly defined or structured program were not included. In addition, studies referring to Chinese medicine were excluded when they did not include a physical exercise component (e.g., acupuncture, herbal remedies, diet, or massage).
Comparison	Control groups of participants with fibromyalgia who received any type of intervention or none, whether pharmacological, some form of physiotherapy, or no treatment at all, regardless of their characteristics (with or without symptoms).	No type of intervention was excluded.
Outcome	Mental health outcomes focusing on anxiety and depression symptoms, assessed using validated instruments such as the BDI [42] or the STAI [43].	Assessments conducted using non-validated instruments.
Time or study type	Systematic reviews of controlled studies, with or without meta-analysis.	Conference posters, scientific communications, and any publications that were not scientific articles or did not report results.

BDI = Beck Depression Inventory, ICD-11 = International Classification of Diseases, 11th Revision, PICOT = Population, Intervention, Comparison, Outcome, and Time or study type, and STAI = State–Trait Anxiety Inventory.

**Table 3:** Study characteristics.

Reference	Study Design	Database Info	Total of RCTs Included	Sample T; EG, CG	Gender M/W/NR(n)	Age
Acosta Carreño et al. (2024) [49]	RS	3 (Scopus, Pubmed y Web of Science). 2019 to 2023	5	182; NR, NR	0/182/0	NR
Cerrillo-Urbina et al. (2015) [35]	Meta but RS	5 (PubMed, Scopus, Science Direct, EBSCO (E-journal, CINAHL, SportDiscus) y The Cochrane Library). 2019 to 2023	2	957; 498, 459	0/957/0	51 mean of years
Cheng et al. (2019) [33]	Meta	3 (PubMed, Medline y Physiotherapy Evidence Database (PEDro)). Until 2019	3	209; 107, 102	29/220/0	36 to 65
de Orleands et al. (2023) [50]	Meta but RS	5 (Web of Science, PubMed, SCOPUS, Cochrane y EBSCO). Until 2022.	1	20; 10, 10	0/0/20	33 to 50
de Souza et al. (2025) [37]	RS	6 (Web of Science, PubMed, EMBASE, SCOPUS, Cochrane y EBSCO). Until March 2024.	4	235; 105, 130	0/235/0	NR
Kelley et al. (2022) [51]	Meta	Based on a previous database that used eight sources, the update for this review was limited to PubMed. Until 14 October 2021.	5	333; 204, 129	NR	41 to 58
Klaps et al. (2022) [32]	RS	4 (PubMed/MEDline, PEDro, Cochrane Library y Web of Science). Until 28 November 2020	3	94; 55, 39	36/53/0	42 to 50
Langhorst et al. (2013) [34]	Meta	5 (Clinicaltrials.Gov, Cochrane Library (CENTRAL), PsycINFO, PubMed y Scopus). Until 31 December 2010	6	306; 152, 154	24/282/0	13.2 to 53.7
McDowell et al. (2017) [25]	Meta	5 (Google Scholar, MEDLINE, PsycINFO, PubMed y Web of Science). January 1995 to June 2016	10	595; 297, 298	9/251/335	42 to 56
Rodríguez-Almagro et al. (2023) [26]	Meta	5 (PubMed (MEDLINE), SCOPUS, Web of Science, CINAHL Complete y PEDro). Until November 2022.	25	NR	NR	NR
Rodríguez-Domínguez et al. (2024) [52]	Meta	7 (PubMed, Cochrane Library, Web of Science, Scopus, PEDro, Dialnet y CINAHL). Until 1 September 2024	7	664; 342, 321	0/664/0	38 to 52
Wu et al. (2025) [31]	Meta but RS	4 (PubMed, Web of Science, Cochrane Library y EBSCOhost (multiple sub-bases)). Until September 2024	1	62; 31, 31	9/53/0	38 to 64
Zhang et al. (2024) [36]	Meta	9 (PubMed, Embase, Scopus, ProQuest, Web of Science, Cochrane, CNKI, WanFang y VIP). Until 1 February 2023.	12	668; 305, 363	184/304/180(4)	10 to 60
Zhang et al. (2025) [53]	Network	8 (Embase, MEDLINE, PubMed, Scopus, Google Scholar, Web of Science, CENTRAL y CNKI). Until March 2022	14	NR	NR	30 to 64

**Table 4:** Results for anxiety.

Reference	Frequency (times/week)	Intensity	Type of Exercise	Time (weeks)	Adherence Rate	Adverse Effects	Comparator	Anxiety	Evaluation Tool	Meta-Analysis
Cerrillo-Urbina et al. (2015) [35]	3	65–75% HRmax	Aquatic exercise	12 to 32	90.9% to 97%	NR	No intervention (1)	Significant differences (2)	STAI (1), FIQ (2)	No meta
de Souza et al. (2025) [37]	2 to 3; NR (1)	Low to high	Stretching exercises supervised via video call (1), in-person aerobic exercise (2), in-person strength training (1), at-home aerobic and strength training (1), aerobic exercise in the pool (1)	4 to 12	12.5% drop out; NR (3)	NR	Unsupervised stretching exercises (1), at-home stretching exercises (1), in-person supervised exercise (1), at-home stretching and isometric strength training (1)	Difference in favor of in-person learning (1)	BDI (3), HADS-D (1), HADS-A (1)	No meta
Kelley et al. (2022) [51]	2 to 7	NR	Land- and pool-based dancing, walking and jogging	10 to 24	0–50% dropouts in aerobic and 0–40.7% dropouts in control	Pain after exercise session (1 participant/1 study), metatarsal fracture (1 participant/1 study), NR (3 studies)	TAU, waitlist	* Aerobic: WMD = −0.77 (95% CI [−1.25, −0.29]), *p* = 0.04	STAI, AIMS, MHI and visual analog scales	Ivhet, IIRD
Klaps et al. (2022) [32]	1 to 3	Low to high	Aerobic exercise (3)	12 to 24	0–50% drop out	NR	Passive (2) and active (1)	A significant improvement was reported in one HIIT group. Two groups that increased intensity showed non-significant results.	HADS (2), BDI (1) and STAI (1)	No meta
McDowell et al. (2017) [25]	1 to 4	NR	Aquatic exercise (5), land-based aerobic exercise (2), land-based multicomponent exercise (1), yoga/mind–body exercise (1), dance (1)	6 to 32	72%	NR	Wait-list control (3), no treatment (3), usual care (2), education (1), Pharmacological treatment (1)	All *: 0.28 (95% CI [0.16–0.40]), *p* = 0.28	STAI (3), FIQ (7), HADS-A (1), MHI-A (1), AIMS-A (1)	Mixed, g Hedges
Rodríguez-Almagro et al. (2023) [26]	NR	NR	Circuit-based exercise and exercise movement techniques	12 to >48	NR	NR	Control	* Circuit-based exercise: −0.37; 95% CI [−0.5, −0.24] *p* < 0.001; * exercise movement techniques: −0.37; 95% CI [−0.66, −0.08] *p* = 0.013	NR	Mixed, d Cohen
Zhang et al. (2025) [53]	1 to 9	NR	Qigong (3), overground exercise (10), aquatic exercise (1)	7 to 24	NR	NR	Wait list (5), education (3), TAU (1), aquatic exercise (4), hydrotherapy (1)	Five of the six non-pharmacological interventions (83.34%) were associated with significant improvements in anxiety compared with usual care, with aquatic exercise showing the greatest effect (P-score = 94.25%; SMD = −1.14), followed by cognitive–behavioral therapy (P-score = 74.47%), while all interventions except education outperformed usual care.	BDI (8), CES-D (2), HAD-D (2), FIQ-D (2), STAI (4), HAD-A (3), FIQ-A (2), PSQI (1)	Mixed, NR

Note: * in favour of intervention.

**Table 5:** Results from depression.

Reference	Frequency (times/week)	Intensity	Type of Exercise	Time (weeks)	Adherence Rate	Adverse Effects	Comparator	Depression	Evaluation Tool	Meta-Analysis
Acosta Carreño et al. (2024) [49]	NR	NR	Spinal stabilization exercises + Kinesio taping (1), Zumba (1), low-intensity physical exercise (1), therapeutic exercise + education on the neurophysiology of pain (1), strengthening exercises (1)	6 to 15	NR	NR	Stabilization exercises (1), aerobic exercises (1), control group (2), therapeutic exercise (1)	No differences between groups (3); the exercise group shows improvement (2)	BDI (1), BDI-II (2), HADS-D (2)	No meta
Cerrillo-Urbina et al. (2015) [35]	3	65–75% HRmax	Aquatic exercise	12 to 32	90.9% to 97%	NR	No intervention (1)	Significative differences (2)	STAI (1), FIQ (2)	No meta
Cheng et al. (2019) [33]	1 to 2	NR	Taichi	10 to 16	NR	No (only personal reasons)	Education (3)	Taichi: −0.49 (95% CI [−0.97, −0.01]), *p* = 0.06	FIQ (1), HADS (1), CES-D (1)	Mixed, SMD
de Orleands et al. (2023) [50]	6	NR	Yoga	4	NR	NR	Pilates (1)	No significative differences	BDI (1)	No meta
de Souza et al. (2025) [37]	2 to 3; NR (1)	Low to high	Video call-supervised stretching exercises (1), in-person aerobic exercise (2), in-person resistance training (1), at-home aerobic and strength training (1), aquatic aerobic exercise (1)	4 to 12	12.5% drop out; NR (3)	NR	Unsupervised stretching exercises (1), at-home stretching exercises (1), in-person supervised exercise (1), at-home stretching and isometric strength training (1)	Significant intra-group difference in favor of the home-based exercise (2); no differences between supervised and unsupervised exercises (2) or when comparing home-based and in-person exercises (1); difference in favor of in-person exercises (1)	BDI (3), HADS-D (1), HADS-A (1)	No meta
Klaps et al. (2022) [32]	1 to 3	Low to high	Aerobic exercise (3)	12 to 24	0–50% drop out	NR	Pasive (2) and active (1)	HIIT showed significant improvement (2); intensity progression showed non-significant results (2)	HADS (2), BDI (1) and STAI (1)	No meta
Langhorst et al. (2013) [34]	1 to 3	NR	Yoga (2), Tai Chi (2), Qigong (2)	4 to 12	64% to 92.80% (NR = 2)	Pain, muscular inflammation and chlorine hypersensitivity	Education (1), delayed treatment control (1), aerobic (1), stretching (1), TAU (1)	All *: −0.49 [−0.76, −0.22], *p* = 0.19	BDI (2), CES-D (1), VAS 0–10 (2), CDI (1)	Mixed, g Hedges
Rodríguez-Domínguez et al. (2024) [52]	2 to 3	40–80% RPM	Strength	3 to 21	101 dropout (EG = 44; CG = 57)	NR	No intervention (3), stretching (3), aerobic (3), relaxation (3)	* All: −0.54; 95% CI [−0.92, −0.16], *p* = 0.005	BDI (3), HADS (4), FIQ VAS (2), CES-D (1) and IDATE (1)	Mixed, NR
Wu et al. (2025) [31]	NR	NR	Qigong Ba-Duan-Jin	12	NR	NR	TAU	* Qiong: SMD = −0.43 (95% CI: −0.62, −0.25), *p* < 0.001	BDI	No meta
Zhang et al. (2024) [36]	1 to 7	Low	Qigong (5), Taijiquan-Tai Chi (2), Baduanjin (2), Wuqinxi (2) and Yijinjing (1)	8 to 12	NR	Save (5), NR (6), shoulder pain and fasciitis (1)	Usual care (3), education (3), medicine (2), active (4), placebo (1)	* Baduanjin: −0.43 (95% CI [−0.77, −0.08]), *p* = 0.015; Qigong: −0.21 (95% CI [−0.46, 0.03]), *p* = 0.086; Tai Chi: −0.31 (95% CI [−1.10, 0.49]), *p* = 0.107; Wuqinxi: −0.36 (95% CI [−1.37, 0.66]), *p* = 0.140; Yijinjing: 0.68 (95% CI [−0.10, 1.46]), *p* = 0.088	BDI (4), HAMD (3), HADS (3), CDI (1) and CES-D (1)	Mixed, g Hedges
Zhang et al. (2025) [53]	1 to 9	NR	Qigong (3), overground exercise (10), aquatic exercise (1)	7 to 24	NR	NR	Wait list (5), education (3), TAU (1), Aquatic exercise (4), hydrotherapy (1)	Six of the seven non-pharmacological interventions (85.71%) were associated with significant improvements in depression, with aquatic exercise showing the greatest effect (P-score = 96.51%; SMD = −1.18), followed by hydrotherapy (P-score = 67.65%), while all interventions except meditation outperformed usual care.	BDI (8), CES-D (2), HAD-D (2), FIQ-D (2), STAI (4), HAD-A (3), FIQ-A (2), PSQI (1)	Mixed, NR

Note: * in favour of intervention.

## Data Availability

No new data were created or analyzed in this study. Data sharing is not applicable to this article.

## Supplementary Materials

The following supporting information can be downloaded at: https://www.mdpi.com/article/10.3390/jfmk11020193/s1, Supplementary Materials S1: excel data of selection, overlap, and AMSTAR-2.
